# Towards Room Temperature Thermochromic Coatings with controllable NIR-IR modulation for solar heat management & smart windows applications

**DOI:** 10.1038/s41598-024-52021-7

**Published:** 2024-02-02

**Authors:** B. S. Khanyile, N. Numan, A. Simo, M. Nkosi, C. B. Mtshali, Z. Khumalo, I. G. Madiba, B. Mabakachaba, H. Swart, E. Coetsee-Hugo, Mart-Mari Duvenhage, E. Lee, M. Henini, A. Gibaud, M. Chaker, P. Rezaee, N. Lethole, M. Akbari, R. Morad, M. Maaza

**Affiliations:** 1 MRD-Tandetron Accelerator & Nanosciences African Network, iThemba LABS-National Research Foundation, P O Box 722, Somerset West, 7129 Western Cape Province South Africa; 2 UNESCO-UNISA Africa Chair in Nanosciences and Nanotechnology, College of Graduate Studies, University of South Africa, Muckleneuk Ridge, P.O. Box 392, Pretoria, 003 South Africa; 3 Physics Department, University of the Western Cape, P.O. Box 1906, Bellville, 7535 South Africa; 4 Faculty of Natural and Agricultural Sciences, Physics Department, University of the Free State, P.O. Box 339, Bloemfontein, 9300 Republic of South Africa; 5 School of Physics & Astronomy, Nottingham University, Nottingham, NG7 2RD7 UK; 6 IMMM, UMR 6283 CNRS, Bd O. Messiaen, University of Le Maine, 72085 Le Mans Cedex 09, France; 7 INRS-Energie et Matériaux, 1650 Lionel-Boulet, Varennes, Québec J3X 1S2 Canada; 8 Department Physics, University of Fort Hare, Alice, Eastern Cape Province South Africa

**Keywords:** Energy science and technology, Materials science, Nanoscience and technology

## Abstract

Solar heat management & green air-conditioning are among the major technologies that could mitigate heat islands phenomenon while minimizing significantly the CO_2_ global foot-print within the building & automotive sectors. Chromogenic materials in general, and thermochromic smart coatings especially are promising candidates that consent a noteworthy dynamic solar radiation Infrared (NIR-IR) regulation and hence an efficient solar heat management especially with the expected increase of the global seasonal temperature. Within this contribution, two major challenging bottlenecks in vanadium oxide based smart coatings were addressed. It is validated for the first time that the NIR-IR modulation of the optical transmission (∆T_TRANS_ = T_(T〈TMIT)_ − T_(T〉TMIT_) of Vanadium oxide based smart coatings can be controlled & tuned. This upmost challenging bottle-neck controllability/tunability is confirmed via a genuine approach alongside to a simultaneous drastic reduction of the phase transition temperature T_MIT_ from 68.8 °C to nearly room temperature. More precisely, a substantial thermochromism in multilayered V_2_O_5_/V/V_2_O_5_ stacks equivalent to that of standard pure VO_2_ thin films but with a far lower transition temperature, is reported. Such a multilayered V_2_O_5_/V/V_2_O_5_ thermochromic system exhibited a net control & tunability of the optical transmission modulation in the NIR-IR (∆T_TRANS_) via the nano-scaled thickness’ control of the intermediate Vanadium layer. In addition, the control of ∆T_TRANS_ is accompanied by a tremendous diminution of the thermochromic transition temperature from the elevated bulk value of 68.8 °C to the range of 27.5–37.5 ºC. The observed remarkable and reversible thermochromism in such multilayered nano-scaled system of V_2_O_5_/V/V_2_O_5_ is likely to be ascribed to a noteworthy interfacial diffusion, and an indirect doping by alkaline ions diffusing from the borosilicate substrate. It is hoped that the current findings would contribute in advancing thermochromic smart window technology and their applications for solar heat management in glass windows in general, skyscraper especially & in the automotive industry. If so, this would open a path to a sustainable green air-conditioning with zero-energy input.

## Introduction

The rise of the global average temperature correlated to climate change has generalized heat islands phenomenon. This latter is becoming a major concern especially with the fast rising rural to urban population exodus. Yet, this singularity was well known for almost a century, it became dominant recently as observed in densely populated cities where excessive temperatures were registered. Such recent elevated & localized temperatures are caused by the important release of anthropogenic heat, and the excess storage of solar radiation within the city compounds. In addition, it is exacerbated by the shortage of green spaces and cool sinks, the lack of effective circulation of air within the city landscape as well as the reduced ability of the emitted infrared radiations to escape in the atmosphere^1^. As summarized by Santamouris et al., Papanikolaou et al.^2–6^, several potential technologies can be used to mitigate such a heat islands phenomenon. Among which; (1) Roof greening, (2) White reflective roofing, (3) Walls greening, (4) Usage of natural heat sinks view of dissipating heat excess, (5) Expanding green spaces, (6) Usage of advanced reflective materials based on nanotechnology additives such as thermochromic paints and coated glass windows^7–9^, and (7) Generalization of the usage of smart windows in both buildings & automotives. This later mitigating cooling technology would not only improve the indoor thermal comfort but minimize the energy consumption due to air–conditioning in addition to the reduction of the CO_2_ footprint.

As per today, ~ 55% of the world’s population lives in urban areas. It is projected that it would reach ~ 2.5 billion by 2050, with ~ 90% of this increase in Asia & Africa. With such an upsurge of the urban population and climate change increase of the average seasonal atmospheric temperature, air-conditioning demand is expected to sky-rocket. The global stock of air conditioners in buildings & automotive sectors is expected to grow up to ~ 5.6 billion by 2050 from the current pool of ~ 1.6 billion today units. The International Energy Agency (IEA)’s projections^10^ for the global energy demand in air-conditioning is expected to triple by 2050. This paramount increase will entail the usage of a significant electricity supply. Henceforth, sustainable technologies are required to minimize the corresponding energy pressure and the related CO_2_ footprint. Chromogenics and/or radiative cooling coatings are potential genuine solutions as a sustainable pathway for green air-conditioning^11–13^.

**Figure 1:**
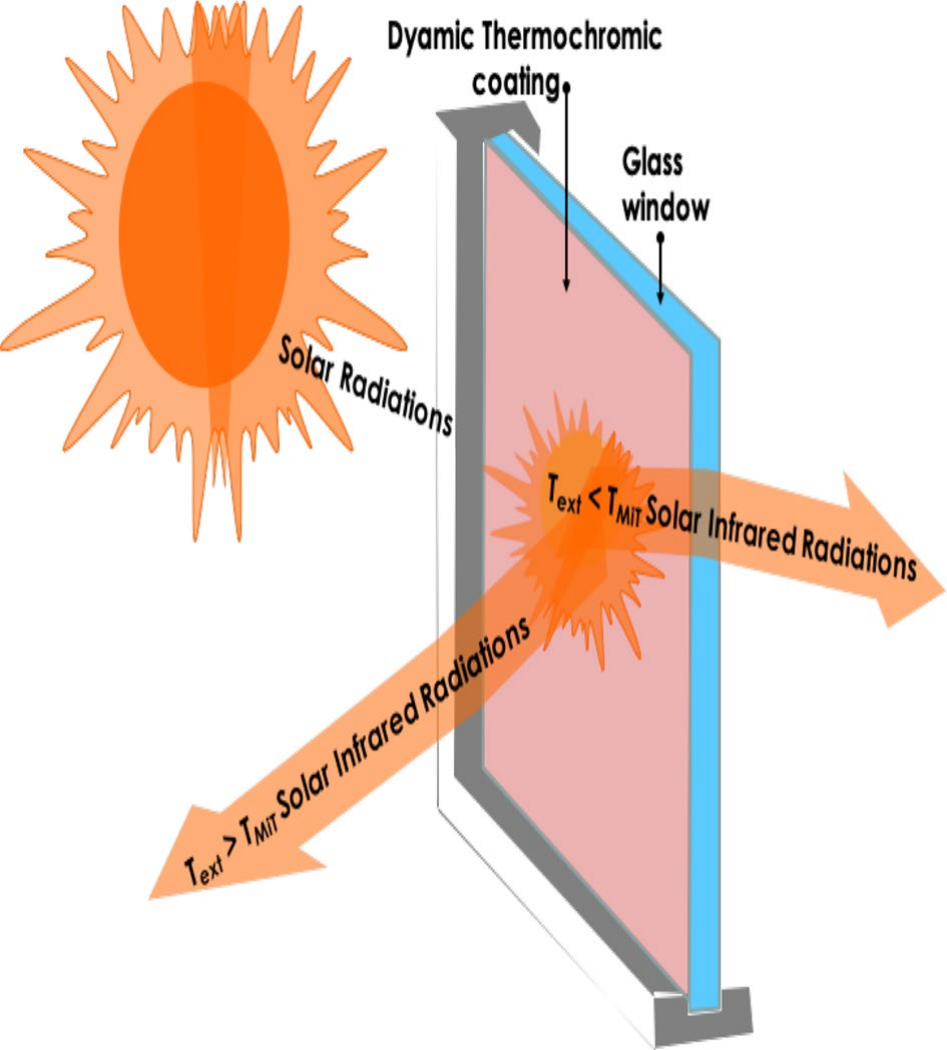
Dynamic solar heat management in VO_x_ based smat window. &Schematic representation of a thermochromic coated glass window,

**Figure 2:**
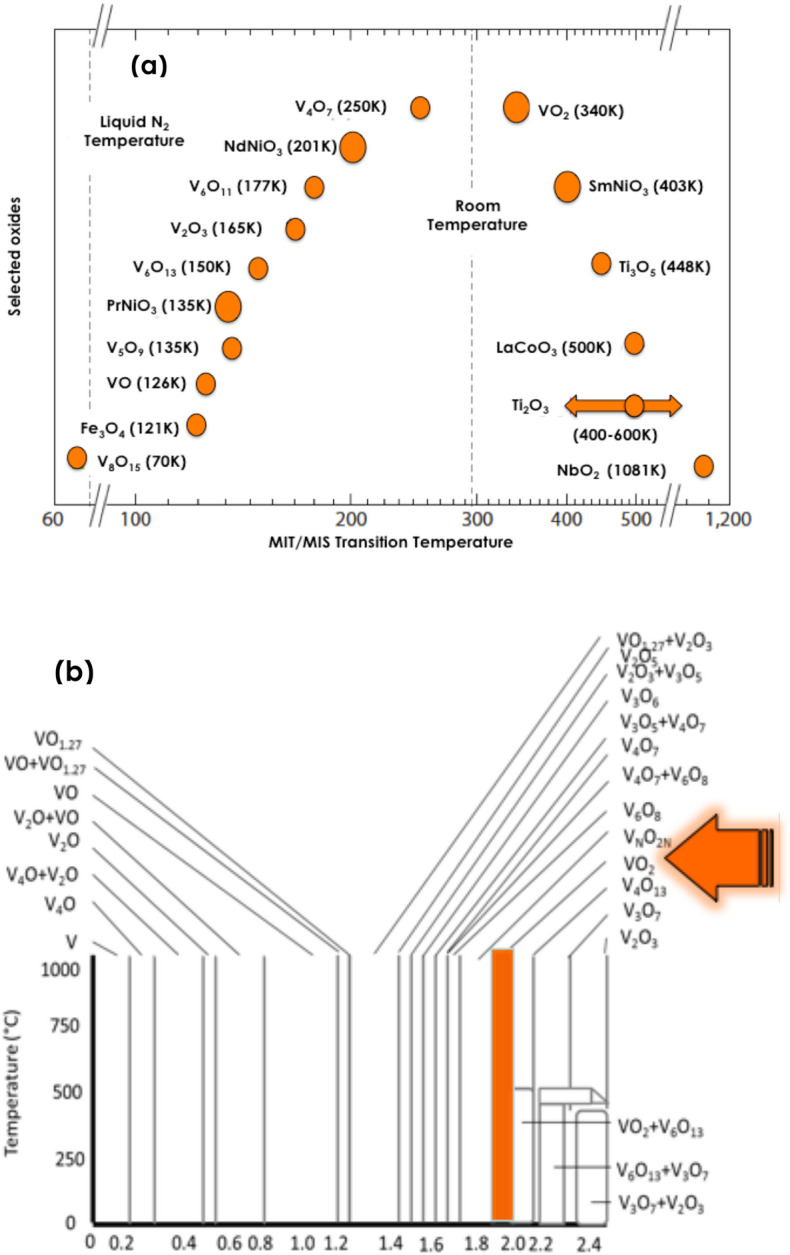
(**a**) Major oxides exhibiting Metallic-Insulator or Metallic-Semiconductor phase transition [Dominated by Vanadium oxides]. (**b**) Temperature-Oxygen Concentration phase diagram of the V–O system.

Among the chromogenic technologies, including electrochromism, and gasochromism, thermochromic nano-coatings for smart windows applications (Fig. 1) have attracted a special interest since the observation of the reversible semiconductor-metallic 1st order transition in Vanadium dioxide (VO_2_) by Morin in 1959^14^. Since then, several oxides were found to exhibit such a 1st order phase transition as displayed in (Fig. 2a)^15–17^. As one can notice, several of these oxides are Vanadium based. Figure 2b reports the corresponding Pressure-Concentration stability phase- diagram of such a rich family of Vanadium oxides correlated to the multivalence of Vanadium; namely V^+5^, V^+4^, V^+3^, V^+2^, V^+1^. Moreover, there are 2 additional large V_x_O_y_ families including the Magnussen (V_n_O_2n+1_) & Wadsley (V_2n_O_5n−2_) layered compounds^15–18^. However, VO_2_ was and is the most investigated compound in view of its sharp 1st order ultrafast phase transition taking place at the vicinity of T_MIT_ ~ 68 °C^14,18,19^ which is close to room temperature and hence the corresponding potential technological applications in solar heat management as intelligent coating for smart windows in the building and automotive sectors in particular^11^.

**Figure 3:**
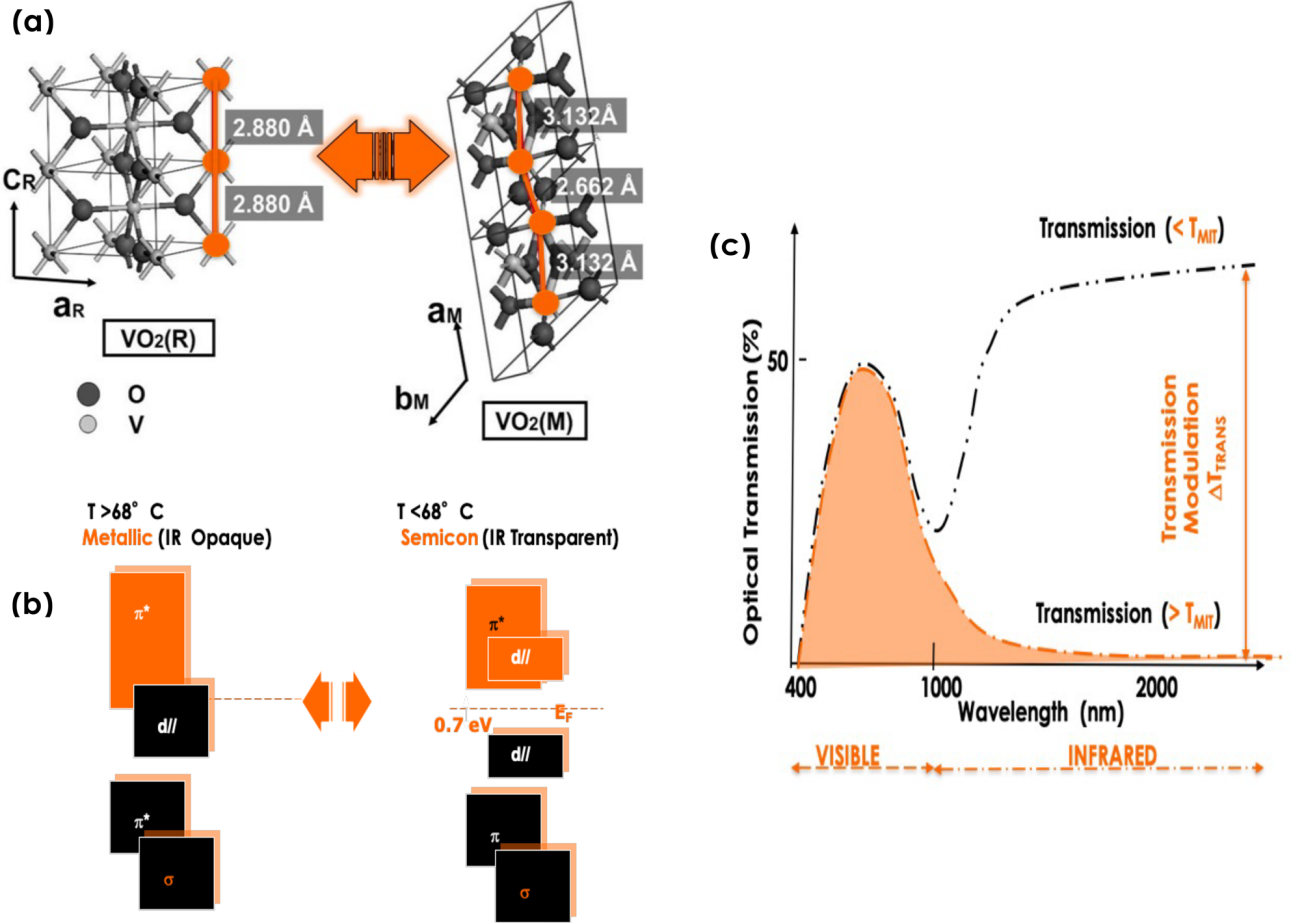
Major properties related to the VO_2_ 1st order phase transition. (**a**) Reversible Monoclinic-Rutile crystallographic phase transition of VO_2_ at the vicinity of T_MiT_ ~ 68 ºC, (**b**) The corresponding reversible electronic phase transition due to the reversible opening-closing of the d band orbital, (**c**) Ideal optical response of a VO_2_ based thermochromic coating; (1) T_VIS_ > 50%), (2) T_MIT_ ~ 25 °C) and primarily, a large modulation in the (NIR) & (IR) spectral range (∆T_TRANS_).

Indeed, and as demonstrated both experimentally and theoretically, VO_2_ exhibits a 1st order reversible phase transition from a semiconductor state to a metallic state & vis-versa upon heating/cooling^11–14^. Such a reversible phase transition which can be stimulated via an external thermal, pressure or optical stimuli is both crystallographic and electronic^18,19^. From crystallographic viewpoint, the low temperature monoclinic VO_2_ structure transits reversibly to a tetragonal phase above T_MIT_ ~ 68 °C (Fig. 3a). Such a femtosecond crystallographic transition is correlated to an electronic transition. This later originates from the splitting of the d// band inducing the creation of a band-gap of the order of 0.72 eV below T_MIT_ ~ 68 °C which closes above (Fig. 3b)^15^.

Besides the thermochromic properties for smart windows applications, VO_2_ was found to be effective in a variety of technological applications. This includes o tunable nano-plasmonics^20,21^, ultrafast optoelectronic gating^22,23^, chemresistors for H_2_ gas sensing^24,25^ and tunable emissivity space coatings for small satellites^26,27^ as well as optical limiting and laser radiations protection coatings^28–30^.

In addition to these multifunctionalities, VO_2_ & related family oxides have raised and impacted substantially several fundamental questions in condensed matter specifically & solid state physics in general. As reported in Table 1, this includes but not limited to: shed-lighting on the sliding twin-domains in self-heated needle-like VO_2_ single crystals^31^, evolution of the Mott transition in chain structure of strained VO_2_ films revealed by coherent phonons^32^, investigation of the solid-state triple point at the metal–insulator transition in VO_2_^33^, Observation of a large non-thermal contribution to picosecond strain pulse generation using the photo-induced phase transition in VO_2_^34^, elucidating on the inhomogeneity of the ultrafast insulator-to-metal transition dynamics of VO_2_^35^, clarifying the transient dynamics of the phase transition in VO_2_ revealed by mega-electron-volt ultrafast electron diffraction^36^, observation of the magnetic-field-induced insulator–metal transition in W-doped VO_2_ at 500 T^37^, demonstration of the reversible phase modulation and hydrogen storage in multivalent VO_2_ epitaxial thin films^38^, Decoupling the Lattice Distortion and Charge Doping Effects on the Phase Transition Behavior of VO_2_ by Titanium (Ti^4+^) Doping^39^, Observation of the photo-induced non-volatile VO_2_ phase transition for neuromorphic ultraviolet sensors^40^ in addition to numerous ultrafast technological proofs of concept^41–43^.

**Table 1:** Selected recent major findings in VO_2_ based systems.

System’s Configuration	Investigated major phenomena & properties	References
VO_2_ Single crystal	Sliding twin-domains in self-heated needle-like VO_2_ single crystals	^31^
VO_2_ Strained thin films	Mott transition in chain structure of strained VO_2_ films revealed by coherent phonons	^32^
Single-crystal VO_2_ nanobeams	Measurement of a solid-state triple point at the metal–insulator transition in VO_2_	^33^
VO_2_ Single crystal	Observation of a large non-thermal contribution to picosecond train pulse generation using the photo-induced phase transition in VO_2_	^34^
VO_2_ Single crystal	Elucidating the inhomogeneity of the ultrafast insulator-to-metal transition dynamics of VO_2_	^35^
VO_2_ Single crystal	Clarification of the transient dynamics of the phase transition in VO_2_ revealed by mega-electron-volt ultrafast electron diffraction	^36^
W-doped VO_2_ thin films	observation of the magnetic-field-induced insulator–metal transition in W-doped VO_2_ at 500 T	^37^
VO_2_ epitaxial thin films	demonstration of the reversible phase modulation & hydrogen storage in multivalent VO_2_ epitaxial thin films	^38^
Ti_x_V_1-x_O_2_ nanopowders	Decoupling the Lattice Distortion and Charge Doping Effects on the Phase Transition Behavior of VO^2^ by Titanium (Ti^4+^) Doping	^39^
VO_2_ Thin films	Photo-induction in non-volatile VO_2_ phase transition for neuromorphic ultraviolet sensors	^40^
VO_2_ ultrathin films on Si_3_N_4_ membranes	Ultrafast X-ray imaging of the light-induced phase transition in VO_2_	^41^
VO_2_ Single crystal	Demonstration of the reversible switching between pressure induced amorphization and thermal-driven recrystallization in VO_2_(B) nanosheets	^42^
ultrathin VO_2_ channels	Validation of the positive-bias gate-controlled metal–insulator transition in ultrathin VO2 channels with TiO2 gate dielectrics	^43^

Yet, the above mentioned technological applications and fundamental focii are directly related to the modulation of the optical transmission of the VO_2_ in the NIR-IR spectral region especially, this latter is the pivotal and central parameter for solar heat management & smart windows applications^10–12,16,17^. As schematically summarized in Fig. 3c, an ideal VO_2_ based thermochromic coating should exhibit the following major characteristics: (1) a good and temperature independent optical transmission in the visible (VIS) spectral range (with a priori a VIS transmission > 50%), (2) a phase transition temperature T_MIT_ close to room temperature (far below the bulk VO_2_ transition of T_MiT_ ~ 68 °C) and primarily, a noteworthy large modulation in the Near Infrared (NIR) and Infrared (IR) spectral range (∆T_TRANS_). This latter is the difference between the optical transmissions below and above T_MiT_; (∆T_TRANS_ = T_(T〈TMIT)_ − T_(T〉TMIT_ > 50%). Although, this later modulation in the NIR & IR spectral range is exceedingly challenging, the lowering of the T_MIT_ and the raising of the VIS optical transmission have been successfully dealt with through an adequate doping (W, Mo, Mg,…) and via the usage of an additional anti-reflection treatment (TiO_2_, ZrO_2_,ZnO,…) respectively^44–48^.

In the pursuit of engineering optimal thermochromic VO_2_ based coatings with a substantial elevated and tunable modulation in the NIR-IR spectral range (∆T_TRANS_ = T_(T〈TMIT)_ − T_(T〉TMIT_), several physical & chemical methodologies were & are used for its deposition^49–61^. Excluding limited cases, the general usage of single and unique layer of VO_2_ was and is the dominating trend in thermochromic VO_2_ based applications. Likewise, in its doped or un-doped form, the VO_2_ based thermochromic coatings suffer in terms of modulation in the Infrared region (∆T_TRANS_). The same situation is faced even in the case of the VO_2_ deposition onto various substrates; amorphous (glass, PET,…)^44,45^ or crystalline (Quartz, Silicon, Mica,…)^46–48,62–73^ in nature. Simply summarized, the VO_2_ based thermochromic coatings suffer drastically in terms of modulation in the Infrared region (∆T_TRANS_) specifically.

**Figure 4:**
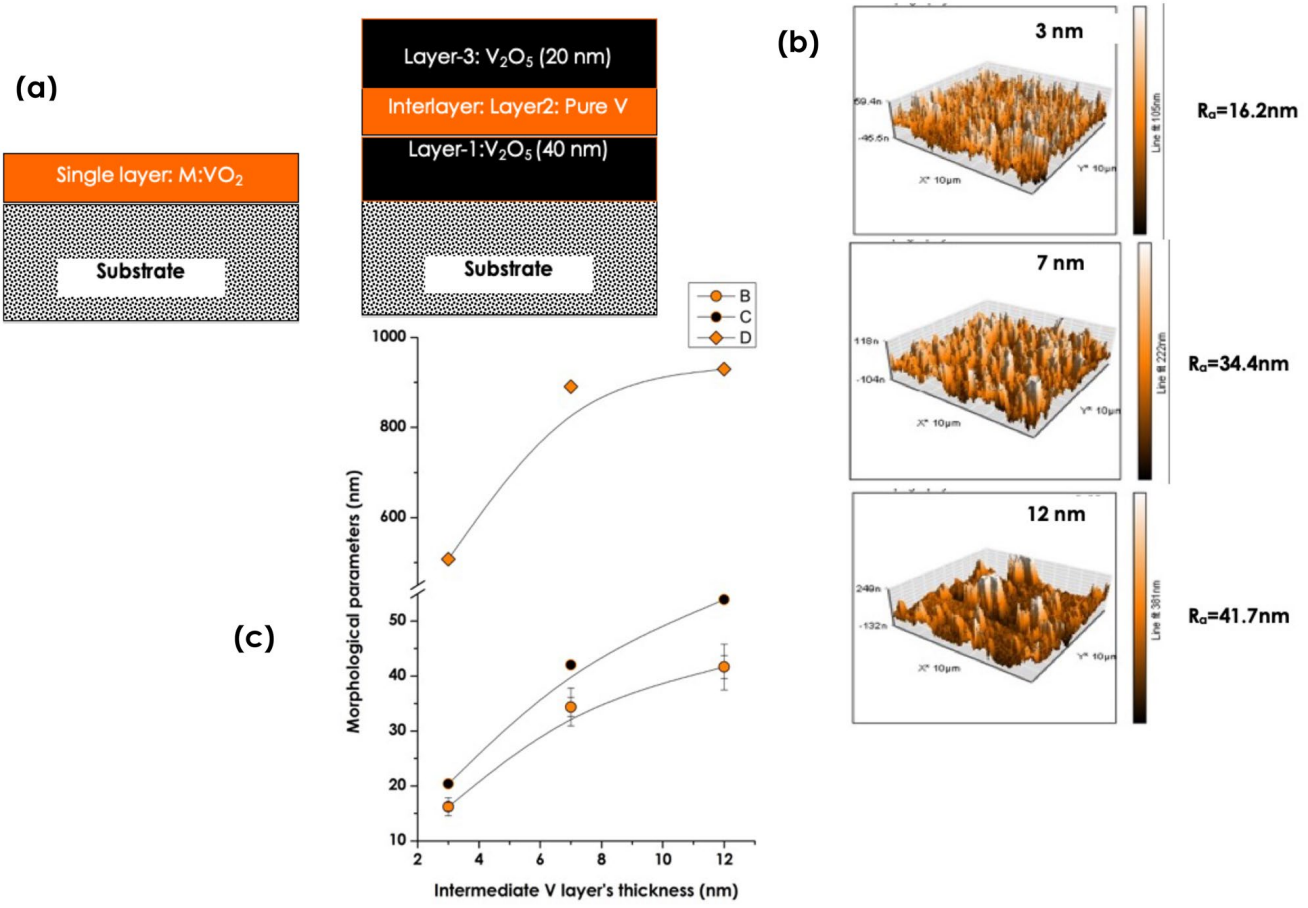
Configurations of the engineered thermochromic coating & its major surface morphological characteristics. (**a**) Standard Single VO_2_ layer configuration & the current considered multi-layered structure consisting of V_2_O_5_(Fixed thickness:20 nm)/pure V (Variable thickness)/V_2_O_5_ (Fixed thickness:40 nm) deposited onto glass substrate, (**b**) Surface Atomic Force Microscopy surface morphology of the 3 multilayered samples, (**c**) Evolution of corresponding average roughness R_a_ (yellow filled circles), the root mean square (R_σ_) (black filled circles), and the average crystallites size (Ø) (yellow filled diamonds).

In this regard, and within the novelty & originality of this contribution, it is validated for the first time that the NIR-IR modulation of the optical transmission (∆T_TRANS_) can be controlled/tuned via an authentic novel approach with a simultaneous drastic reduction of T_MIT_. This latter original approach consists of using a multilayered configuration instead of the standard single VO_2_ layer approach as illustrated in Fig. 4a. In addition, it requires the usage of a Magnussen V oxide such as V_2_O_5_ and not the standard pure VO_2_. For the validation of such a NIR-IR modulation control with a simultaneous drastic reduction of T_MIT_, a tri-layer stack consisting of V_2_O_5_/V/V_2_O_5_ deposited onto glass substrate was considered. Within such a multi-layered stack of V_2_O_5_/V/V_2_O_5_ onto borosilicate glass substrate, the V_2_O_5_ layers’ thickness is fixed and that of the interlayer of pure V is varied followed by an optimal post annealing.

Yet original in terms of approach, it is to be stressed that this research work has been inspired by several partial contributions including but not limited to Long et al.^74^, Pellegrino et al.^75^, Wang, Miller et al.^76^, Zheng et al.^77^, Zhou et al.^78^, Han et al.^79^, Zhao et al.^80^ while fostering the previous work by Sfundo et al.^81^. As per the scientific & patented published literature, and as it was highlighted previously, other multi-layered nano-structures were proposed but none allowed a significant control of the NIR-IR modulation with a significant decrease of the T_MIT_ simultaneously. Table 2 summarizes such multilayered nano-structures.

**Table 2:** Major published VO_2_ based multilayered coatings.

Configuration of the multilayered system	Synthesis technique	Transition temperature (^°^C)	References
V_2_O_3_/VO_2_	Reactive magnetron sputtering	72.0	Long et al.^74^
VO_2_/TiO_2_	Pulsed Laser Deposition	63.5	Pellegrino et al.^75^
ITO/VO_2_/TiO_2_	Reactive magnetron Sputtering	52.0	Miller and Wang^76^
TiO_2_/VO_2_/TiO_2_	Medium frequency reactive magnetron sputtering	61.5	Zheng et al.^77^
SiO_2_/VO_2_	Spin coating	61.0	Wang et al.^82^
V_2_O_3_/V (7 nm)/V_2_O_3_	Electron beam Evaporation With a non optimised annealing	27.9	Sfundo et al.^81^
V_2_O_3_/V(12 nm)/V_2_O_3_	Electron beam Evaporation With a non optimised annealing	36.9	Sfundo et al.^81^
VO_2_/Au/VO_2_	Electron beam evaporation	66.9	Zhou et al.^78^
V_2_O_5_/V/V_2_O_5_	Electron beam evaporation	high-temperature coefficient of resistance	Han et al.^79^
SiO_2_-TiO_2_/VO_2_/ TiO_2_	Spin coating	36.9	Zhao et al.^80^
WO_3_/VO_2_/WO_3_	Reactive magnetron sputtering	54.5	Long et al.^83^
V_2_O_5_/V(7 nm)/V_2_O_5_	Electron beam evaporation	27.5	Current work
V_2_O_5_/V12nm)/V_2_O_5_	Electron beam evaporation	37.5	Current work

The specific objectives of this contribution are as follows.

1. Validation of thermochromism in V_2_O_5_/V/V_2_O_5_ sandwich nanostructures,
2. Validation of the tunability/control of the modulation in the NIR-IR spectral range (∆T_TRANS_ = T (T_〈TMIT_) − T_(T〉TMIT)_,
3. Validation of the tunability/control of the transition temperature T_MIT_,
4. Validation of the effectiveness of Alkaline doping in T_MIT_ tenability.

Comparatively to the previous published literature as recapitulated in Table 2, the current research contributes in advancing the subject of Vanadium oxide based thermochromism via two major milestones; (1) the crucial role of the intermediate V layer within the V_2_O_5_/V/V_2_O_5_ sandwich nanostructures in terms of the tunability of both the optical modulation in the NIR-IR spectral range (∆T_TRANS_ = T(_T〈TMIT_) − T(_T〉TMIT_), and that of the transition temperature T_MIT_, (2) the role of the alkaline ions doping originating from the borosilicate substrate. As per our best knowledge, none of the above mentioned parameters were investigated so far.

## Materials and methods

### Samples preparation

All chemicals & substrates used in these experiments are of high chemical grade (from Sigma-Aldrich &/or Alfa-Aesar). Following a sequential procedure, multi-layered films of V_2_O_5_/V/V_2_O_5_ were deposited by e-beam evaporation using V_2_O_5_ powder and V metal material targets and borosilicate glass substrates (10 × 10 × 2.5 mm^3^). The substrates were cleaned in an ultrasonic bath with methanol and de-ionized water for ~ 20 min prior to deposition. All substrates were dried with pressurized 100% pure N_2_ gas before being loaded into the deposition chamber, which was already loaded with highly pure vanadium (V) and vanadium pentoxide (V_2_O_5_) targets in separate crucibles. The multi-layered stacks were deposited at an initial chamber vacuum pressure of ~ 10^−6^ mbar and an evaporation rate of ~ 0.24 nm/s. The V_2_O_5_ & pure V layers’ thicknesses were monitored with a standard crystal monitor. The thickness of the 1st layer (bottom) of V_2_O_5_, was fixed to ~ 40 nm, while the thickness of the intermediate layer (V layer) was varied within a defined range of 3–12 nm at specific values of 3, 7 and 12 ± 0.2 nm. The thickness of the 3rd (top layer) of (V_2_O_5_) was fixed at 20 nm. These thicknesses were chosen based on a preliminary set of computations, which suggested that the film be chosen in such a way that sufficient diffusion of oxygen into that inter-layer would take place in addition of an additional interfacial stress/strain; with both layers allowing O atoms to diffuse into the V intermediate layer. All prepared samples were annealed for 120 min in a vacuum of ~ 10^−6^ mbar at the temperature of 500 °C.

### Samples characterization

The surface morphology of the samples was investigated using Atomic Force Microscopy (AFM) in non-contact mode complemented by an in-depth/volume morphology studies using a Field Emission Scanning Electron Microscopy (FE-SEM, Jeol JSM-7800F). The crystal structure of the various samples was examined with a Bruker AXS D8 Advanced X-ray diffractometer, which was outfitted with a copper X-ray tube (λ = 0.15406 nm) and operated at 40 kV and 40 mA with data collection in the Θ–2Θ configuration within the angular range of 15–60 Deg (in steps of 0.01 Deg). For the elemental analysis and depth profiling, Auger Electron Spectroscopy (AES) and Time of Flight Secondary Ion Mass Spectrometry (ToF-SIMS) were used. The optical measurements within the spectral range of 250–2500 nm were carried out in transmittance mode (normal incidence) with a Cary 5000 UV–VIS–NIR spectrometer equipped with a controllable heating stage with a heating/cooling rate of 5 °C/min within the temperature range of 20–90 °C.

## Results and discussions

### Morphological investigations: AFM & FE SEM

Figure 4b displays the Atomic Force Microscopy of the various samples. The surfaces are relatively rough suggesting, a priori, the crystalline nature of the multi-layered stack samples or at least the top surface of layer 3 (V_2_O_5_ top layer). Table 3 summarizes the corresponding values of the average roughness (R_a_), the root mean square (R_σ_), the average height (H) as well as the average crystallites size (Ø). Accordingly, thicker is the intermediate V layer, higher are the various parameters 〈R_a_〉, 〈R_σ_〉, 〈H〉 and 〈Ø〉. Yet limited to 3 values of the intermediate V layer’s thickness, it can be safely concluded that the effect of this latter (i.e. the intermediate V layer’s thickness) is of a prime effectiveness in view of the variations of Table 3 & Fig. 4c.

**Table 3:** AFM characteristic parameters of thermal annealed multi-layered films.

Sample identification	Intermediate V layer thickness (nm)	Average roughness 〈R_a_〉 (nm)	Root Mean Square roughness 〈R_σ_〉 (nm)	Average height H 〈H〉 (nm)	Average Grain size 〈Ø〉 (nm)
V_2_O_5_/3nmV/V_2_O_5_	3	16.21	20.39	1.04	507.82
V_2_O_5_/7nmV/V_2_O_5_	7	34.37	42.04	2.36	890.62
V_2_O_5_/12nmV/V_2_O_5_	12	41.65	53.93	2.47	929.76

**Figure 5:**
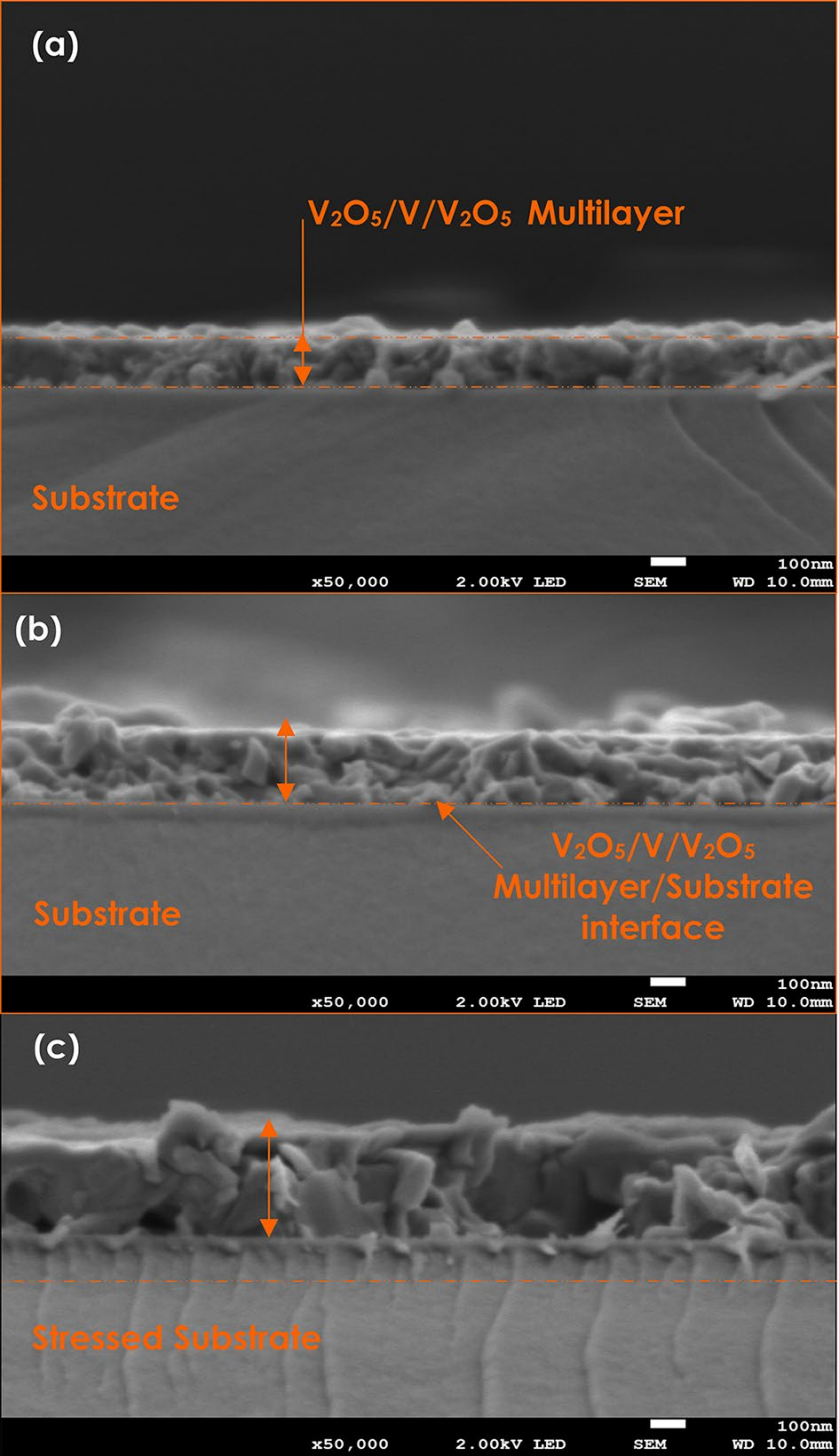
In volume cross-section of the various multi-layered V_2_O_5_ (20 nm)/pure V (Variable thickness)/V_2_O_5_ (40 nm) deposited onto glass substrate & their Auger elemental depth profiling.

Figure 5 displays the FESEM edge cross-section of the various multi-layered samples. As one can notice, in each and all of the samples, it is not possible to distinguish the various layers of the stacks V_2_O_5_/V/V_2_O_5_ deposited onto the borosilicate glass substrates. Indeed, excluding the crystal-clear net interface with the substrate, there are no sharp interfaces between bottom layer 1 (V_2_O_5_) and the intermediate V layer as well as this latter and top layer 3 (V_2_O_5_). This is likely a signature of a noteworthy interfacial diffusion within both interfaces of the intermediate layer of V and top/bottom surrounding layers of V_2_O_5_. This observation seems to be supported by the observed various nano-crystals distributed isotropicaly within the transversal direction of the stack throughout the 2 interfaces of Fig. 5. If one considers the O and V atomic/ionic radii, it could be, safely concluded that Oxygen is prospectively to diffuse from the O rich regions i.e. from layers 1 & 3 of V_2_O_5_ towards the O poor region i.e. towards the pure V intermediate layer. Last but not least, one can observe several cracks within the substrate of sample 3 i.e. (12 nm V intermediate layer thickness). These cracks seem to be initiated from layer 1-substrate interface and propagating towards the inner section of the substrate. These cracks are likely to originate from a substantial stress/strain relaxation at the interface layer 1/substrate at least. As a preliminary pre-conclusion of this section, one could cautiously conclude on the elevated interfacial diffusion within the interfaces (top & bottom) surrounding the intermediate layer of pure Vanadium. Such a significant interfacial diffusion would not only affect the chemical composition profile of the multi-layered stack but also the strain/stress distribution.

### Elemental analysis & depth profiling: Auger analysis

**Figure 6:**
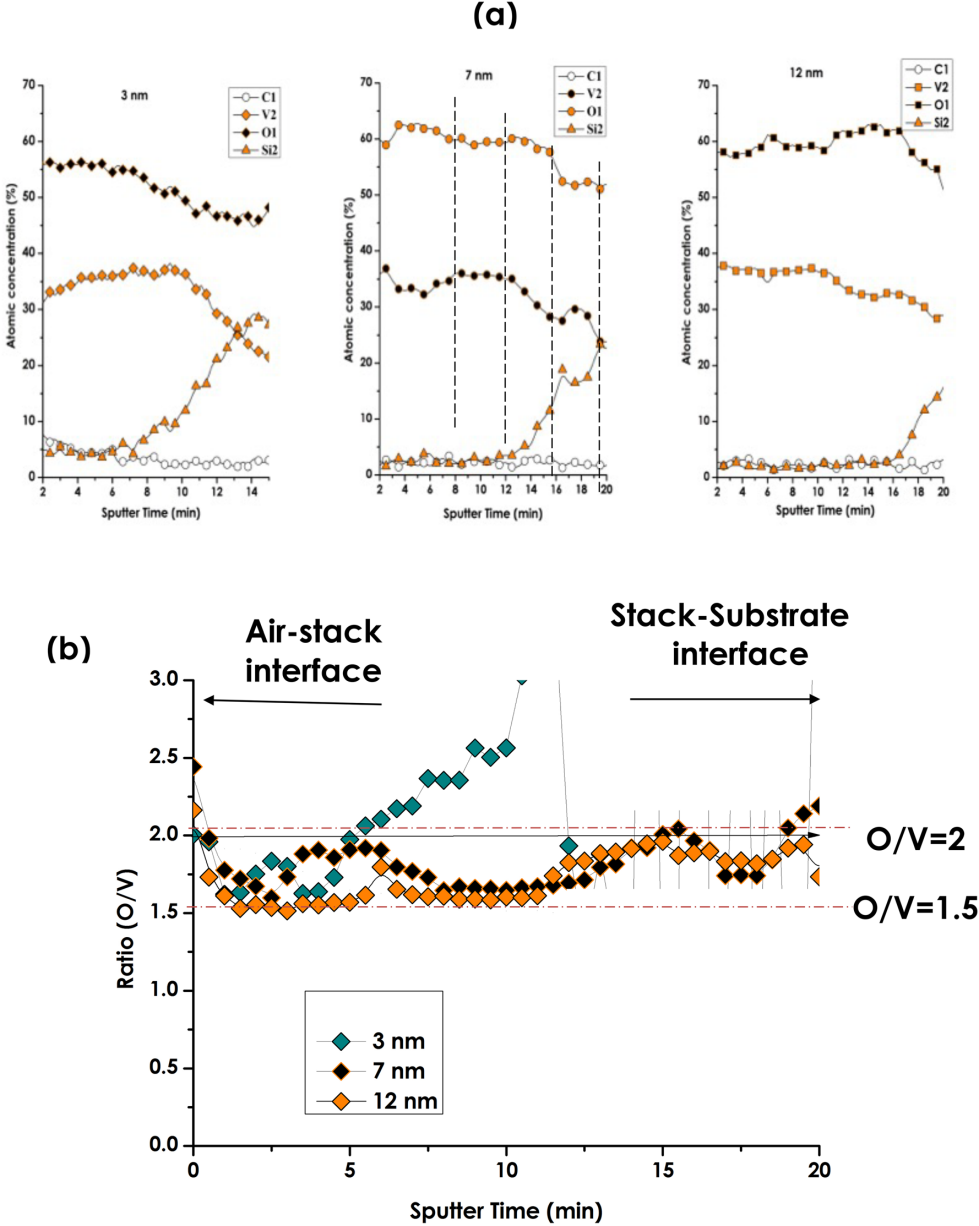
(**a**) The various multi-layered V_2_O_5_(20 nm)/pure V(Variable thickness)/V_2_O_5_ (40 nm)deposited onto glass substrate corresponding Auger chemical profiling of C, V, O & Si, and (**b**) the corresponding O/V ratio.

Figure 6a displays the elemental depth profiling of Carbon (C), Oxygen (O), Vanadium (V) and Silicon (Si) of the 3 samples obtained via Auger spectrometry investigations. The C originates from the Carbon coating layer deposited onto the surface sample initially for charge removal. By contrast, Si originates from the borosilicate glass substrate. This latter consists of 70–80 wt% SiO_2_ of amorphous SiO_2_ in addition to other oxides (7–13 wt% of B_2_O_3_, 4–8 wt% Na_2_O, K_2_O, and 2–8 wt% of Al_2_O_3_^84^. As one can notice, the Si diffusion is mainly localized at the substrate-1st layer of V_2_O_5_ in the 3 multi-layered stacks independently from the intermediate V layer’s thickness. Hence, one could, a priori, conclude that the 1st layer of V_2_O_5_ might acted as a barrier minimizing the diffusion of Si & Al from the borosilicate substrate towards the multi-layered stack.

By contrast, If the O & V concentration depth profiles of Fig. 6a are considered, one can crystal clearly notice the presence of oxygen through-out the transversal direction of the 3 stacks. This is in support of the significant interfacial diffusion mentioned formerly, especially the O diffusion from the surrounding V_2_O_5_ layers towards the intermediate V layer.

Likewise, the O & V concentration depth profiles seem substantially correlated while in phase in the case of samples 2 (V_Intermediate-layer_ = 7 nm) and 3 (V_Intermediate-layer_ = 12 nm) by opposite to sample 1 (V_Intermediate-layer_ = 3 nm). Figure 6b reports the corresponding depth variation of the O/V ratio. Subsequently, and within the bar error of ± 5%, the average value of the O/V ratio seems increasing quasi linearly for sample 1 (V_Intermediate-layer_ = 3 nm) while fluctuating between 1.5 and 2 for sample 2 (V_Intermediate-layer_ = 7 nm) and sample 3 (V_Intermediate-layer_ = 12 nm). Henceforth, it can safely be concluded that in the 2 thicker stacks, the dominating phase or phase are within the V_2_O_3_ & VO_2_ families from stoichiometric viewpoint and/or phases under significant strain/stress (if one considers the observed cracks relaxation in the FESEM images of Fig. 5c).

### *Alkaline doping *via* substrate-stack interfacial diffusion & depth profiling: SIMS analysis*

Because of the small ionic radius of alkaline ions and their low activation energy, It is established that they diffuse relatively fast within the substrate surface via established exchange mechanisms^84,85^. In the case of Na^+^, its low activation energy Q_a_ & its elevated diffusion constant D_0_ (D = D_0_e − Q^a^, activation energy of Q_a_ = 1.36 eV and D_0_ = 3.12 × 10^−4^ m^2^) fovours its diffusion both within the bulk and the surface of the glass substrate^86,87^. Hence, it is necessary to depth profile the alkaline ions. For such Secondary Ions Mass Spectrometry (SIMS) analysis were carried out.

**Figure 7:**
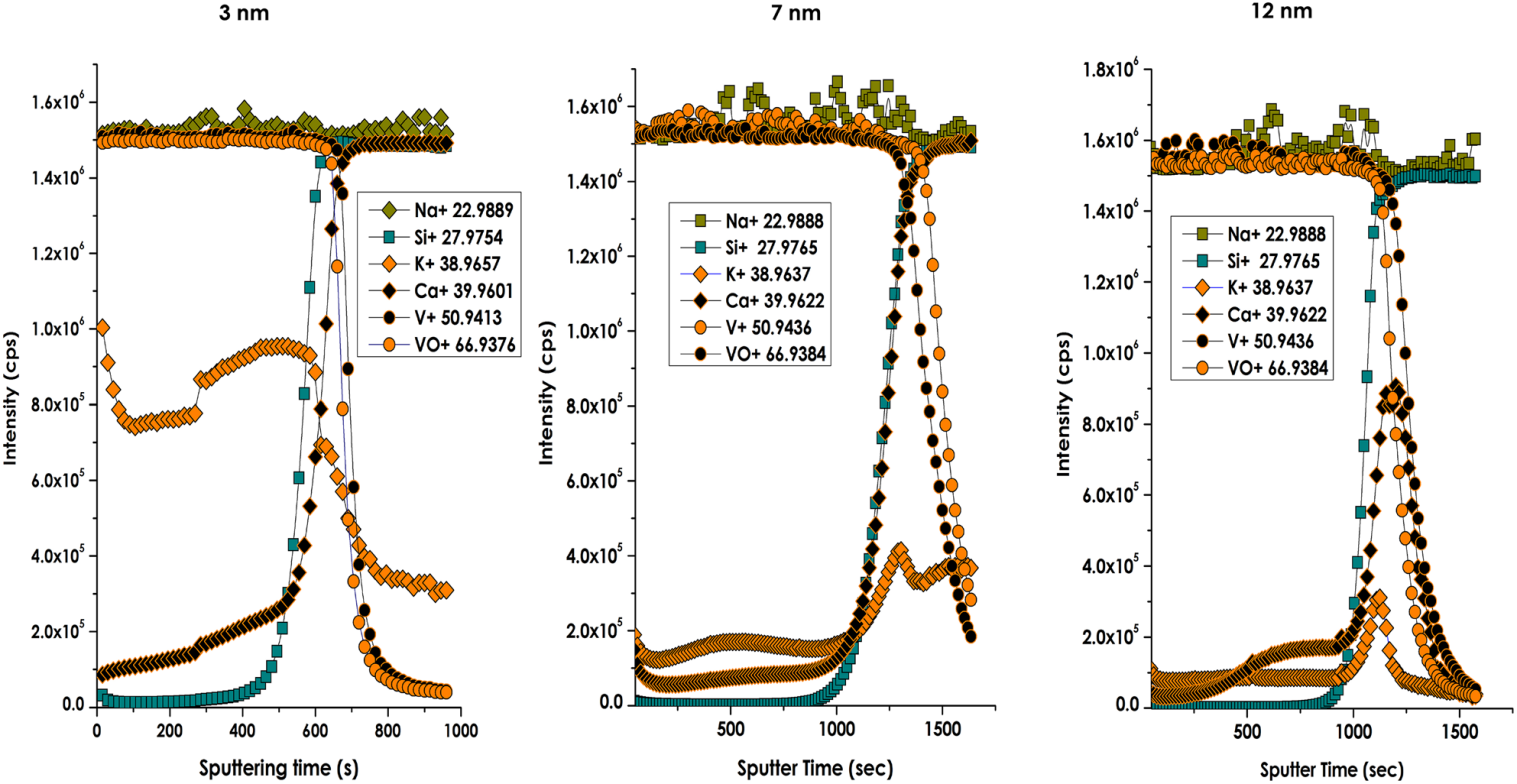
In-depth profile O/V ratio variation derived from Secondary Ion Mass spectrometry (SIMS) elemental depth profiling of various halides (Na^+^, K^+^, Ca^+^), Silicon Si^+^ and Vanadium (V^+^) as well as VO^+^ of the various multi-layered stacks of V_2_O_5_/V/V_2_O_5_ deposited onto borosilicate glass substrate for various intermediate Vanadium layer’s thickness; 3 nm, 7 nm and 12 nm.

Figure 7 displays the SIMS elemental depth profiling of several ions proper to the stack (V^+^, VO^+^) and those alkaline ions from the borosilicate substrate (Na^+^, Ca^+^ & K^+^). These latter explicit ions were targeted in view of the chemical composition of the borosilicate glass substrate (consisting of SiO_2_: 72.5%, Na_2_O: 13.7%, CaO: 9.1%, KO: 12% MgO: 4.2%,…)^70,84,86,87^. Once again, the distribution profiles of V^+^, VO^+^ are alike throughout the multi-layered stack samples and the stack-substrate interface. This matching depth distributions of V^+^ & VO^+^ is in an ample support of the O diffusion observed in the previous Auger profiles. The Si profiles exhibit a heavy-side type variation for each & all samples with a nearly zero counts within the stack in support of a very weak if not a no diffusion of Si originating from the substrate. This latter behaviour is also in accordance with the previous Auger observations. Contrasting with such a Si limited interfacial diffusion is the alkaline ions; namely Na^+^, Ca^+^ and K^+^. All of them display a long-range diffusion from the borosilicate glass throughout the stacks with Na^+^ followed by K^+^ and Ca^+^ up to the air-stacks interface. Such an prominent extended diffusion is likely due to the small ionic radius (Ø_Na+_ ≈ 0.102 nm, Ø_K+_ ≈ 0.138 nm, Ø_Ca+_ ≈ 0.118 nm),and the low activation energy of the concerned alkaline and henceforth their elevated diffusion coefficient (D_oNa+_ ≈ 0.330 × 10^−9^ m^2^/s, D_oK+_ ≈ 1.960 × 10^−9^ m^2^/s, D_oCa+_ ≈ 0.79310^−9^ m^2^/s) by contrast to Mg & Si as well as to V. Nevertheless, there is a crystal-clear difference between the depth distributions of Na^+^, Ca^+^ & K^+^. Relatively to Na^+^, Ca^+^ profiles, the profile of K^+^ is singular. While its depth distribution is inhomogeneous for stack 1 (intermediate V_Int-layer_ = 3 nm), it is very low and nearly constant throughout the V_2_O_5_/V/V_2_O_5_ stacks 2 & 3 i.e. (intermediate V_Int-layer_ = 7 & 12 nm). Besides, the K^+^ profiles exhibit a net localized peak at the interface substrate-stacks 2 & 3. While such an accumulation at the substrate-multi-layered stacks interface is not understood, the constant diffusion within the multi-layered stacks up to the air- multi-layered stacks interface would likely affect the thermochromic properties if any. Indeed, considering the K^+^ & Ca^+^ concentrations within the stacks, this indirect doping would likely affect the thermochromic properties of the multi-layered stacks, especially the transition temperature as in the case of doping with various Materials including W, Mo, Mg. Cr, Ti, Nb,Cs, Sn, F, among others was reported within the literature^88–97^

### Crystallographic properties: texture & crystallites orientations

**Figure 8:**
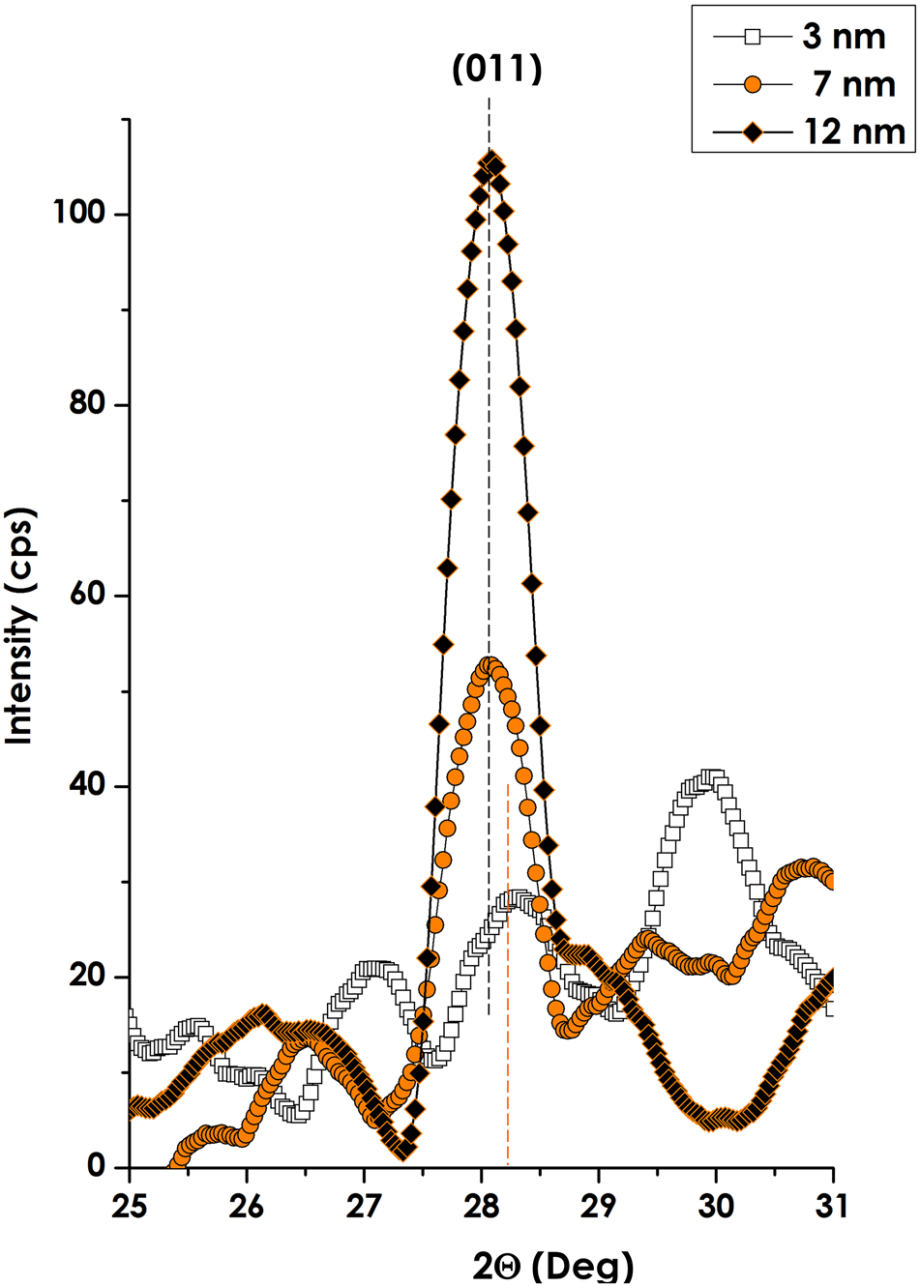
Crystallographic properties the various multi-layered V_2_O_5_(20 nm)/pure V (Variable thickness)/V_2_O_5_ (40 nm)deposited onto borosilicate glass substrate.

Figure 8 reports the room temperature X-ray diffraction pattern of the 3 multilayered stacks of V_2_O_5_/ V /V_2_O_5_ onto borosilicate glass substrate within the Θ–2Θ diffraction configuration. The pattern was limited to the angular region of 25–31 Deg. Such an angular restriction is due to the fact there were no diffraction signatures below and above 25 Deg respectively. On the other hand, one can markedly distinguish the (011) main Bragg diffraction peak of the monoclinic VO_2_ thermochromic active phase which for the bulk or non-strained VO_2_ thin films generally is centred around 2Θ ~ 27.85 Deg. Nevertheless, those observed on the investigated samples are displaced toward higher values by at least ~ 1 Deg (samples 2 & 3). Such an angular shift can only be ascribed to a significant compressive strain/stress on the (011) reticular planes. The relative compression on such a set of reticular atomic planes is relatively high, of the order of ∆d_011_/d_011_ ~ ∆Θ_011_/tan Θ_011_ ≈ 11 & 17% for samples corresponding to intermediate V layer of 7 and 12 nm. On the other hand, the one corresponding to the thinnest intermediate V layer i.e. 3 nm seems to be under a far higher strain/stress in view of the larger angular shift of almost ~ 1.73 Deg. It is to be mentioned that this sample’s XRD profile exhibits, rather, a rich diffraction pattern with 3 consecutive potential low intensity Bragg peaks centred approximately at the vicinity of 27.07, 28.29 and 29.90 Deg. This set of low intensity peaks may correspond to a sub-structure within the 1^st^ sample i.e. (V_Intermediate-layer_ = 3 nm), if any. To shed-light correctly on the nature of such a rich diffraction pattern, it would require, a priori, synchrotron type investigations. More accurately, grazing incidence x-rays diffraction studies^98–100^. One can point out to the absence of the Bragg diffraction peaks of pure Vanadium and V_2_O_5_ including the major most intense ones; V(100), 2Θ ~ 42.172 Deg) & single phase V_2_O_5_ (001), 2Θ ~ 20.251 Deg. As a pre-conclusion of this crystallographic investigation study, it is safe deducing on the significant strain/stress on the nanocrystals and their atomic reticular plans especially those of (011) orientation.

### Optical properties: NIR-IR modulation tunability & control

**Figure 9:**
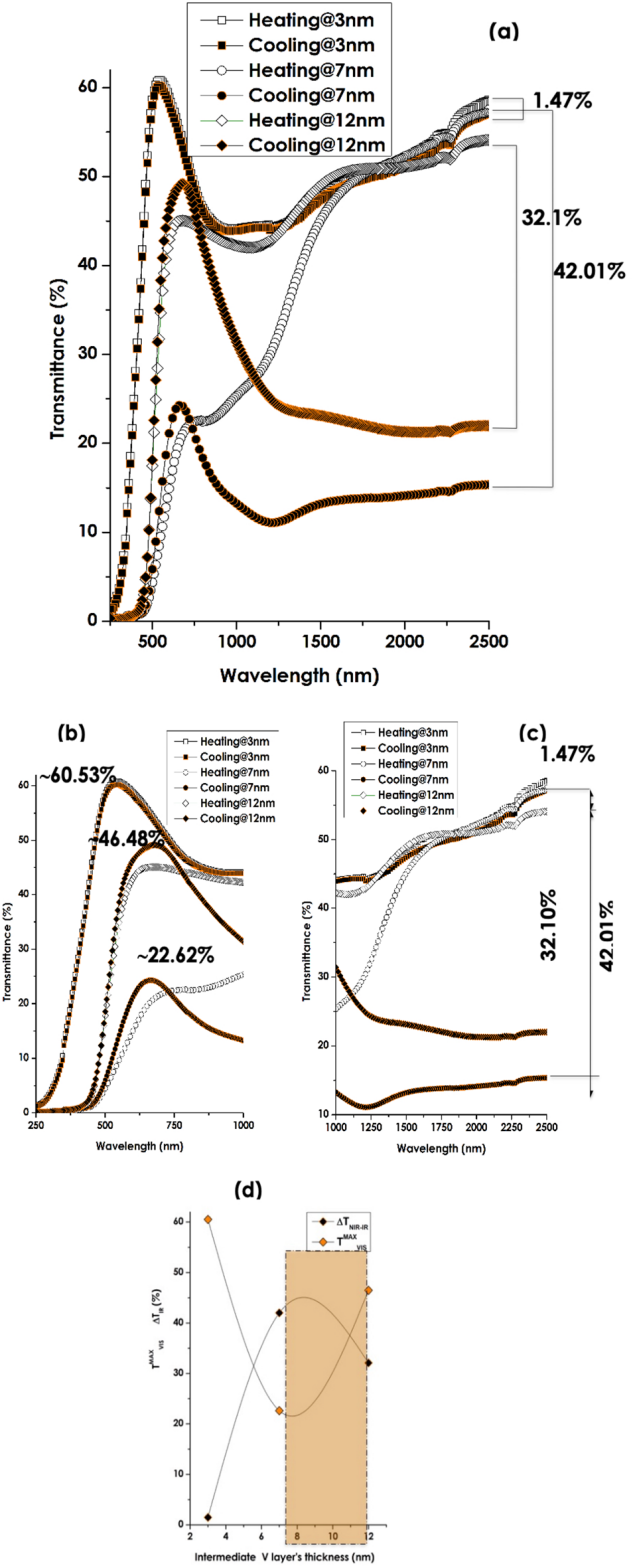
Optical response of the various multi-layered V_2_O_5_(20 nm)/pure V(Variable thickness)/V_2_O_5_ (40 nm)deposited onto borosilicate glass substrate. (**a**) Their room temperature XRD pattern. (**b**) Their corresponding optical transmissions at 25 °C and 70 oC within solar spectrum range of 250–2500 nm. (**c**) Their corresponding optical transmissions at 25 °C and 70 °C within NIR-IR spectral range of 1000–2500 nm and (**d**) their corresponding optical transmissions at 25 °C and 70 °C within VIS spectral range of 250–1000 nm.

Figure 9 reports the pivotal results of this contribution. It displays the experimental optical transmission profiles of the 3 multi-layered stacks of V_2_O_5_/V/V_2_O_5_ onto borosilicate substrate within the solar spectral range of 250–2500 nm below and above the transition temperature T_MIT_ ~ 68 °C. More precisely at 25 °C and 70 °C. Further down, they are labelled as cooling and heating respectively. As one can notice in Fig. 9a, while all of the 3 multi-layered stacks exhibit a net thermochromic signature with a crystal-clear dependence on the intermediate Vanadium layer’s thickness in terms of T_VIS_ as well as the NIR-IR modulation ∆T_TRANS_. This latter is about 1.47%, 42.01% & 32.10% for V_2_O_5_/3nmV/V_2_O_5_, V_2_O_5_/ 7 nm V/V_2_O_5_, and V_2_O_5_/12 nm V/V_2_O_5_ samples respectively. Accordingly and within this multi-layered configuration of V_2_O_5_/V/V_2_O_5_/glass substrate, it is, therefore, safe to conclude that the targeted control/tunability of the NIR-IR modulation ∆T_TRANS_ versus the intermediate V layer’s thickness is validated.

Figure 9b,c display the zoom on the NIR-IR (1000–2500 nm) and VIS (250–1000 nm) solar spectral ranges. In addition to the modulation in the NIR-IR, the transmission in the VIS spectral range is also affected. The corresponding maximum of the optical transmission 〈T_VIS_〉 is about 60.53%, 22.62% and 46.48% for V_2_O_5_/3 nm V/V_2_O_5_, V_2_O_5_/7 nm V/V_2_O_5_, V_2_O_5_/12 nm V/V_2_O_5_ samples respectively. By contrast to single VO_2_ thermochromic coatings, the wavelength corresponding to the maximum of transmission in the VIS spectral range is varying with the intermediate V layer’s thickness too. The spectral position of such a maximum of transmission λ^Max^_VIS_ is centered approximately at 528.7, 682.6, and 665.1 nm for the V_2_O_5_/3nmV/V_2_O_5_, V_2_O_5_/7nmV/V_2_O_5_, V_2_O_5_/12 nm V/V_2_O_5_ samples respectively.

Figure 9d summarizes graphically Table 4 and reports on the evolution of the optical transmission in the VIS; 〈T_VIS_〉 and the optical transmission modulation in the NIR-IR ∆T_TRANS_ (∆T_TRANS_ = T_(T〈TMIT)_ − T_(T〉TMIT_). This figure seems indicating that 〈T_VIS_〉 and ∆T_TRANS_ variations versus the thickness of the intermediate V layer, are in opposition of phase within the considered configuration of V_2_O_5_/V/V_2_O_5_/ onto borosilicate glass substrate. This seems indicating that there is still room for optimization of ∆T_TRANS_ with a sort of trade-off between 〈T_VIS_〉 and ∆T_TRANS_ (shaded region of Fig. 9d).

**Table 4:** Variation of the major optical parameters versus the intermediate V layer’s thickness; Maximum VIS Transmission wavelength 〈λ_VIS_〉, Maximum VIS Transmission wavelength 〈T_VIS_〉, and the NIR-IR modulation ∆T_TRANS_ (∆T_TRANS_ = T _(T〈TMIT)_ − T_(T〉TMIT_).

Sample identification	Intermediate V layer Thickness (nm)	Maximum VIS Transmission wavelength 〈λ_VIS_〉(nm)	Average VIS Transmission 〈T_VIS_〉, (%)	Maximum Transmission T _(T〈TMIT)_ (%)	Minimum Transmission T _(T〉TMIT)_ (%)	Transmission Modulation (∆T_TRANS_ (%)
1-V_2_O_5_/3nmV/V_2_O_5_	3	528.7	60.53	58.58	57.11	1.47
2-V_2_O_5_/7nmV/V_2_O_5_	7	682.6	46.48	54.02	21.92	32.1
3-V_2_O_5_/12nmV/V_2_O_5_	12	665.1	22.62	57.33	15.32	42.01

In addition to the morphological & interfacial diffusion aspects, the major findings so far identified were quantified in terms of the visible optical transmission T_VIS_ and specifically the NIR-IR optical modulation ∆T_TRANS_.

In light of the various interfacial diffusion phenomena and the observed elevated interfacial stress/strain, it is necessary to shed-light on the effective transition temperature of the investigated multi-layered stacks and its evolution versus the intermediate Vanadium layer’s thickness if any. This later, if it does should be diffusional alkaline dopant or/and stress–strain related in view of the non-negligible large lattice mismatch between the V_2_O_5_ and V layers. Concerning this second aspect of crystalline lattice mismatch, this latter would likely induce a noteworthy interfacial stress/strain. This has been evidenced previously through the substantial shift of the (011) Bragg peak observed in the X-rays diffraction spectrum of Fig. 8. This interfacial stress–strain would likely affect the transition temperature of the stack as was reported in the literature by various authors^42,44,64,74^. Likewise, the observed interfacial diffusion of various alkaline ions (K^+^, Na^+^ & Ca^+^) from the borosilicate substrate throughout the multi-layered would prospectively act as potential dopants and hence would impinge on the transition temperature. Such doping with various Materials including W, Mo, Mg. Cr, Ti, Nb, Cs, Sn, F, among others was reported within the literature^88–97^

**Figure 10:**
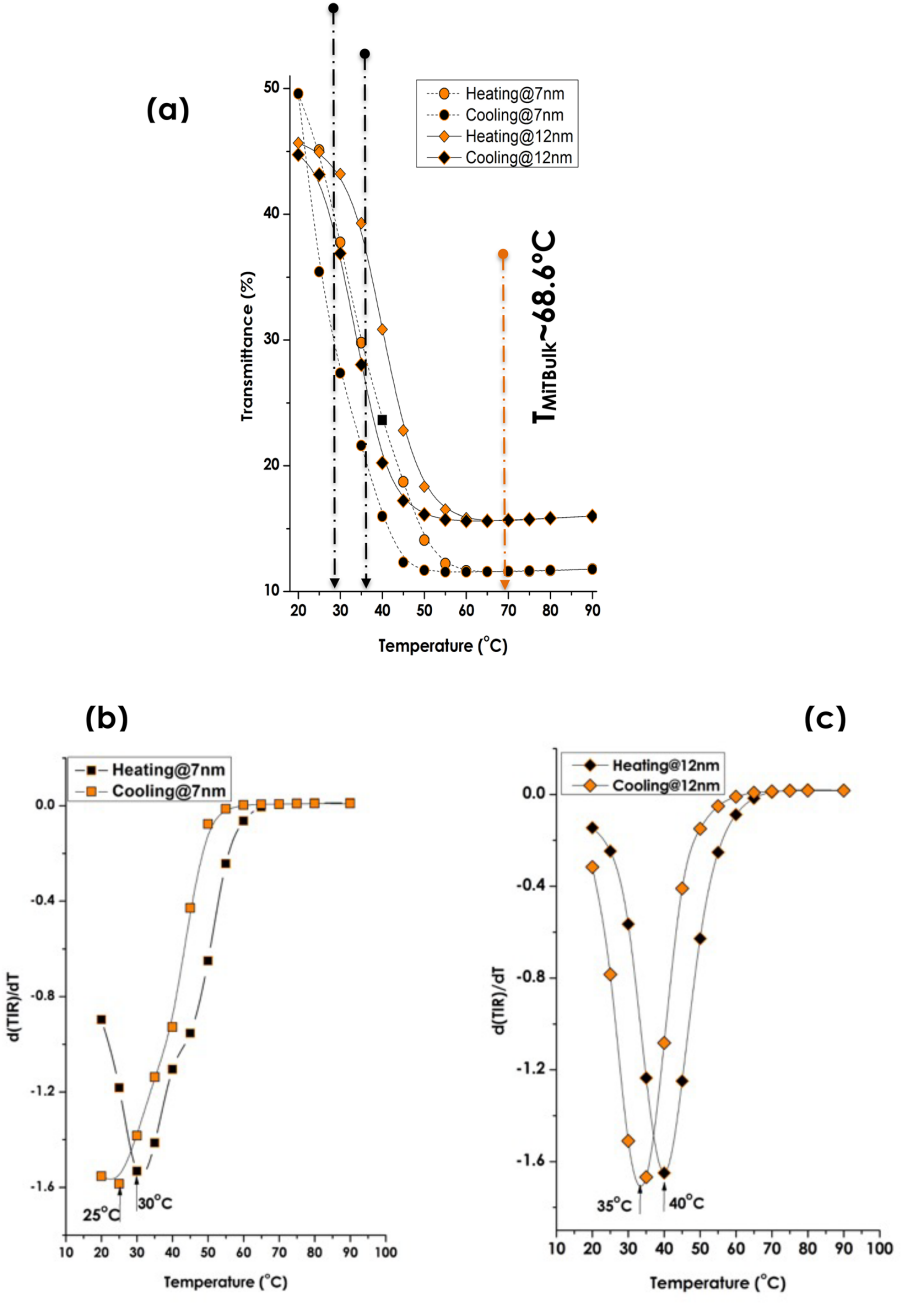
Thermochromic signatures & transition temperatures of the various multi-layered V_2_O_5_(20 nm)/pure V(Variable thickness)/V_2_O_5_ (40 nm)deposited onto borosilicate glass substrate. (**a**) Their optical transmission at 2500 nm during the cooling & heating cycles. (**b**) The derivative of the optical transmission at 2500 nm during the cooling & heating cycles of sample 2 (Intermediate V layer thickness = 7 nm). (**c**) The derivative of the optical transmission at 2500 nm during the cooling & heating cycles of sample 3 (Intermediate V layer thickness = 12 nm),

Henceforth, hysteresis measurements were carried out on the various multi-layered stack samples as shown in Fig. 10a. This latter displays the standard hysteresis of the optical transmission versus temperature for a fixed wavelength (2500 nm) for the multi-layered stack corresponding to the intermediate Vanadium layer‘s thickness of 7 and 12 nm. The hysteresis for the 3 nm V was inconsequential and thus omitted. It can be noticed that the width of the hysteresis for both 7 & 12 nm V layer’s thickness are almost similar in order of δT ~ 7 °C. This value is relatively smaller than the generally reported hysteresis of single VO_2_ thin films which is of the order of δT ≥ 10 °C. The low value of such a hysteresis (δT ~ 7 °C) might be due to the initial large density of nucleating defects during the films growth as observed and concluded by Zhang et al.^101^.

To estimate the corresponding transition temperatures, the standard derivative approach is used. Figure 10b,c display the corresponding derivative hysteresis profiles. The average of the corresponding minima are 27.5 °C and 37.5 °C for the V_2_O_5_/V/V_2_O_5_ multi-layered stacks with the intermediate Vanadium layer’s thickness of 7 and 12 nm respectively. This substantial lowering of the T_MIT_ which is the second prominent finding of this contribution is likely to be ascribed to the above mentioned interfacial diffusion, stress–strain and/or alkaline ions doping.

As a pre-conclusion, one might consider with provision that the investigated V_2_O_5_/V/V_2_O_5_ stacks (with 7 & 12 nm V layer’s thickness) onto borosilicate glass substrate behave as an hypothetic thermochromic V_x_O_y_ phase which exhibits a thickness dependent NIR-IR ∆T_TRANS_ with a lower phase transition temperature of T_MiT_ = 27.5 or 37.5 ºC while under a stern interfacial stress/strain and an alkaline doping. This conjugated doping & stress/strain effects seem to be the most plausible major parameter if one considers the extensive published literature on the subject^102–116^.

Firstly, Tselev et al., and several other authors reported on the prime role of the stress/strain effects in Vanadium dioxide nanocrystals^102–106^. These studies illustrated that a complete picture of the phase transitions in single-crystalline and disordered VO_2_ structures can be drawn only if both ferroelastic and metal–insulator strain effects are taken into consideration.

Secondly, similar stress/strain related behaviour was observed by Nagashima et al.^107,108^. It was found that the stress relaxation affects drastically the transport properties of strained VO_2_ epitaxial thin films grown on TiO_2_ (001) single crystal. When varying the film thickness ranging from 10 to 30 nm, there were no significant changes on the crystal structures. On the other hand, increasing the film thickness resulted in a drastic change on the transport properties including emerging multi-steps in the metal–insulator transition as well as an increase of the resistivity. The discrepancy between the observed crystal structure and the transport properties was related to the presence of nano-scaled line cracks due to thermal stress. It was concluded that controlling thermal stress relaxation rather than the stress due to the lattice mismatch is critical to the intrinsic nature on the transport properties of strained VO_2_ epitaxial thin films.

Thirdly, and likewise, Mathevula et al.^109^, reported that the interfacial stress between VO_2_ thin films deposited onto highly crystalline natural substrates of mica gain considerably in crystallinity without any annealing. This unexpected crystalline growth was observed even with the drastic lattice mismatch between mica & VO_2_ deposited thin films. In this later case, the VO_2_-Mica substrate mismatch induced even a textured VO_2_ films’ crystallographic orientation with a variation of 4 orders of magnitude of the electrical resistance upon the MiT transition with a relatively small hysteresis of about δT ~ 7 °C.

Fourthly and most importantly, a similar behaviour was observed in V_2_O_5_ thin films by Ramana et al.^108^. In this latter case, the V_2_O_5_ thin films were deposited by pulsed-laser deposition and were investigated for their surface-structure evolution in relation to the growth temperature. The deposition was made onto various substrate materials and in the wide range of substrate temperatures, 30–500 °C, keeping the oxygen partial pressure at 100 mTorr. The results gave a consistent picture of the evolution of vanadium oxide film surface morphology and microstructure in terms of growth, behaviour, shape, and distribution of the grains making up films. Their grain size, surface texture, and external morphology of V_2_O_5_ thin films were found to be highly sensitive to the substrate temperature while the effect of substrate material characteristics is dominant only at higher temperatures. The grain size varied in the range 50–600 nm and the surface roughness in the range 9–20 nm with the increasing substrate temperature for crystalline vanadium oxide thin films. Within this study, it was concluded that strain stress relaxation via thermal annealing permit the growth of highly crystalline V_2_O_5_ thin films.

### Computational-modelling studies

As pointed out previously If one considers the O and V atomic/ionic radii, it could be, safely concluded that Oxygen is likely to diffuse from the O rich regions i.e. from layers 1 & 3 of V_2_O_5_ towards the O poor region i.e. towards the pure V intermediate layer. In addition to the experimental confirmation (Fig. 6), this claim can be confirmed by comparing the energy gained from forming VO_2_ from bulk V and O_2_ (in the gas phase) by the energy required to create a double O vacancy in the V_2_O_5_ lattice. The DFT simulations indicate that ~ 7.45 eV are gained when forming VO_2_, while the energy required to create a double Oxygen vacancy in V_2_O_5_ is about ~ 5.92 eV. Therefore, the diffusion of O from V_2_O_5_ into the middle layer and forming VO_2_ is an energetic favourable process.

**Figure 11:**
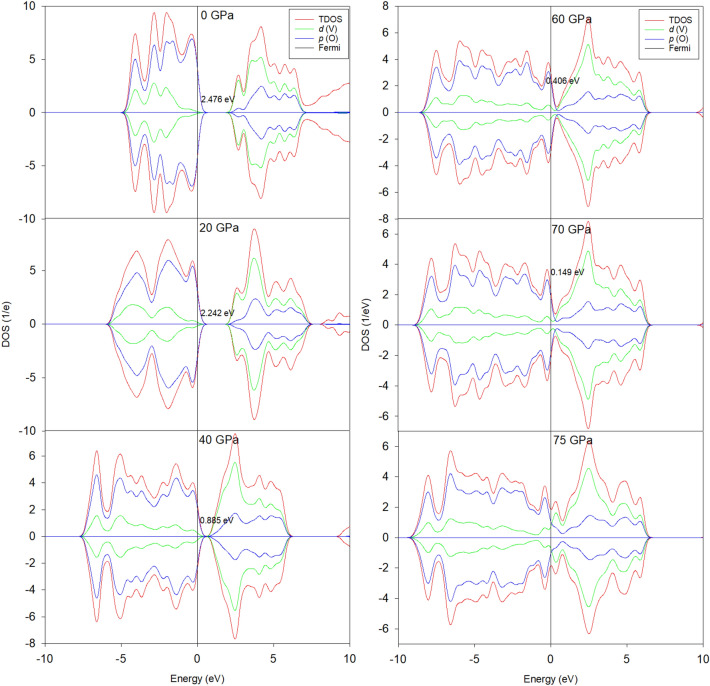
Total and partial density of states (DOS for V_2_O_5_ under pressure (20, 40, 60, 70 & 75 GPa).

**Figure 12:**
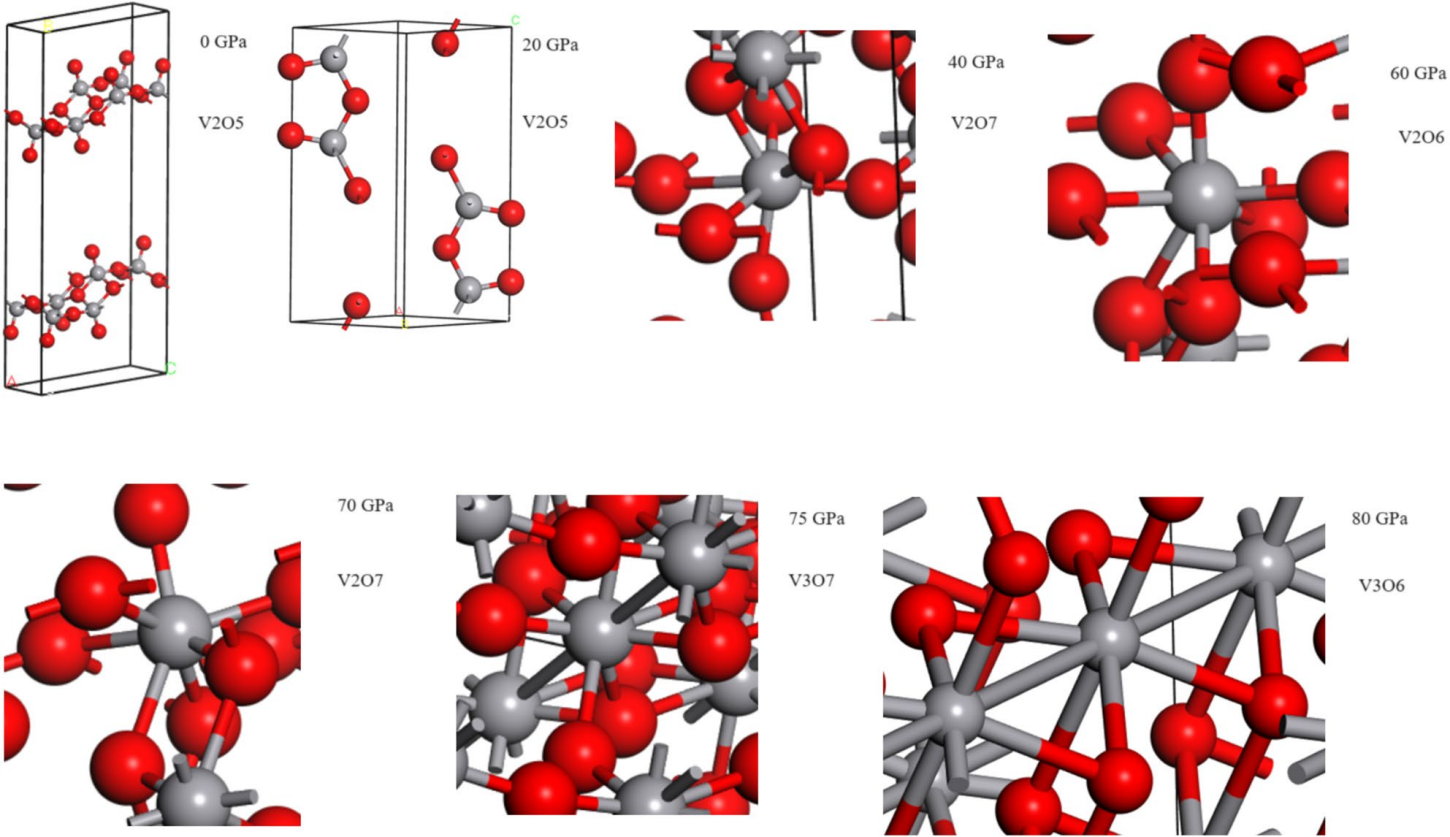
Structural coordination of V_2_O_5_ under various pressures.

To sustain or sap the stress/strain effects on the observed thermochromic properties specifically, the change in structure and electronic properties of V_2_O_5_ under the pressure were investigated using DFT^61^. Such studies are summarized in Figs. 11 and 12. The calculated density of states shows that the energy band gap around the Fermi level shrinks from 2.476 eV at 0 GPa and vanish at ~ 75 GPa. Hence, the material shows insulator behaviour characteristic at low pressure and semiconductor to metallic behaviour at higher pressures. Henceforth, one can conclude that pressure affects significantly the electronic conductivity of the V_2_O_5_ material at relatively excessive elevated values. These extreme pressures are exceedingly elevated to those observed in XRD of Fig. 8. Accordingly, it is safe to conclude that the observed decrease of the transition temperatures of Fig. 10 is unlikely to be interfacial strain/stress/pressure driven.

**Figure 13:**
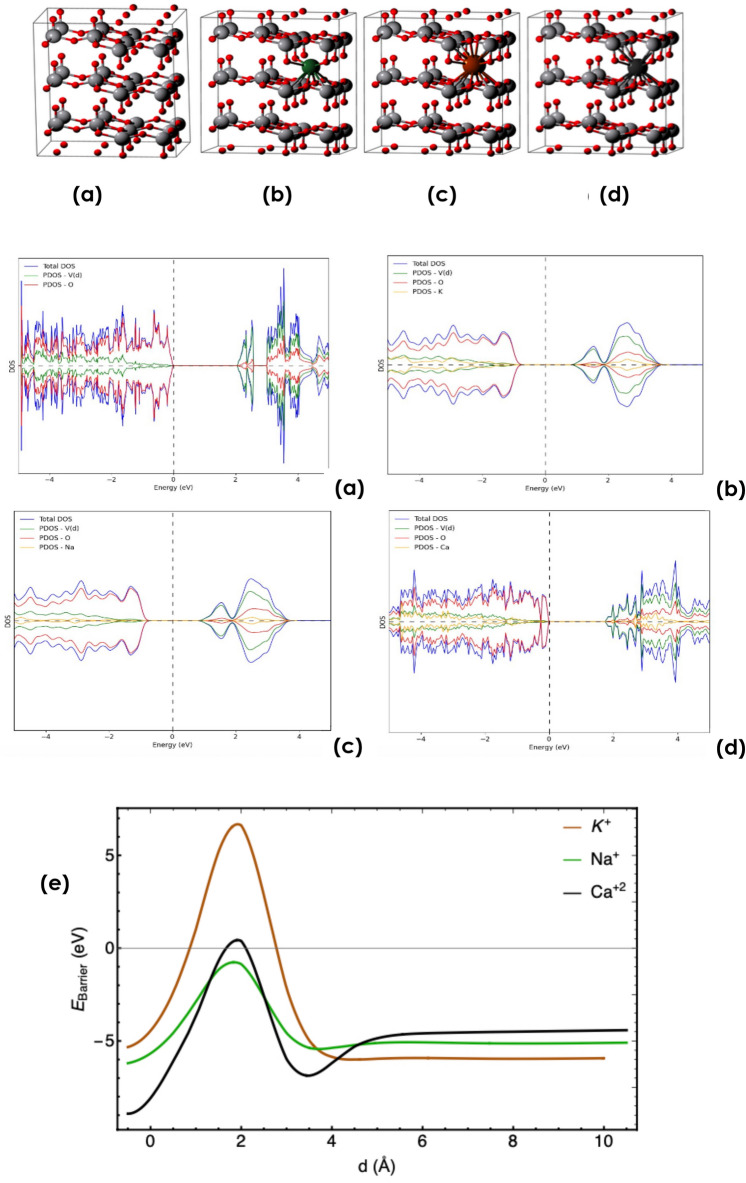
The optimized $2\times 3\times 1$ supercell of (**a**)—$\alpha$V_2_O_5_ (**b**) Na^+^/ $\alpha$—V_2_O_5_ (**c**) K^+^/ $\alpha$—V_2_O_5_ (**d**) Ca^+2^/ $\alpha$—V_2_O_5_. DOS for (**a**) pristine and (**b**) K/—$\alpha$V_2_O_5_, (**c**) Na/—$\alpha$V_2_O_5_, & (**d**) Ca/—$\alpha$V_2_O_5_ for the lowest-energy insertion sites. Positive and negative DOS values indicate the spin up and down, respectively. Energies are relative to the Fermi energy of each system. The partial densities of states for the different atomic species are indicated in green (vanadium), red (oxygen), and yellow (K/Na/Ca ion scaled by a factor of 100 for better visibility) in every panel, (**e**) The barrier energy for ion intercalation into the V_2_O_5_ slab.

In line with the SIMS observations, Na^+^, Ca^+2^ & K^+^ insertion into $\alpha$-V_2_O_5_ was investigated using DFT performed in Quantum-Espresso Package^117,118^ with the plane wave basis set and the PBE functional^117–119^. A plane-wave cut-off energy of 1200 eV and a Monkhorst–Pack k-mesh of $2\times 1\times 1$^120^ was chosen for all calculations. All structures first relaxed with a convergence threshold on forces of 0.01 eV/Å. The electronic structure calculations was performed using the tetrahedron method and a finer k-mesh of $3\times 2\times 2$ points was utilized. For V d states, a rotationally invariant Hubbard correction was used with (U-J) = 3.5 eV^121^ and also a Grimme (D2) dispersion correction with pair-wise interactions was applied on the oxygen atoms^122^ to account the weak van-der Waals (vdW) interactions within the layered vanadium oxide. A single K/Na/Ca ion inserted into a $2\times 3\times 1$ supercell of $\alpha$-V_2_O_5_ corresponding to a A_0.06_ V_2_O_5_ where A is K/Na/Ca ion. Once the most favourable insertion sites with the lowest energy were identified for all ions (Fig. 13a–d), the change in electronic structure upon insertion into these preferred locations was conducted. The density of states (DOS) of pristine $\alpha$-V_2_O_5_, K^+^/V_2_O_5_, Na^+^/V_2_O_5_, and Ca^+2^/V_2_O_5_ are plotted displaying significant band gaps of approximately 2 eV with zero net magnetic moment. The conduction bands consist of mainly V d orbitals, while the valence band have mostly O 2p orbital contribution. It is obvious that the insertion of alkaline ions shifts the Fermi energy.

The insertion energy is calculated by subtracting the energy of V_2_O_5_ and metal (A) from the ionated V_2_O_5_,

$${\text{E}}_{{{\text{ins}}}} = {\text{E}}({\text{A}}/\alpha - {\text{V}}_{2} {\text{O}}_{5} ) - {\text{E}}(\alpha - {\text{V}}_{2} {\text{O}}_{5} ) - {\text{E}}({\text{A}}).$$

The optimized structures’ corresponding insertion energies for K^+^, Na^+^ & Ca^+2^ are − 6.5, − 6.8 & − 13.3 eV, respectively which indicate the V_2_O_5_ is an effective host for ion insertion.

Since the insertion energies are relatively high, the energy barrier for intercalation of the alkaline ions into the surface of V_2_O_5_ using DFT was also investigated. The results are plotted in Fig. 13e**,** where d denote the distance from the slab in Å. Although all energies show a big barrier at around 2 Å, the intercalation energy for Na^+^ ion is always negative which means that it is favorable. For K^+^, if the energy required to pass the barrier at 2 Å is provided, the ion can intercalate between the layers easily. This trend indicates that the V_2_O_5_ is an effective host for ion insertion in general and those originating from the surface of the borosilicate substrate specifically.

As a pre-conclusion, and if one considers the intercalation aspect of Fig. 13a–d as well as the corresponding DOS distributions, one could safely conclude that the variations of the transition temperatures are likely to be caused by the alkaline ions doping and unlikely by the interfacial stress/strain. As a follow up & a foresight future investigations, the luminescence properties of the currently investigated samples will be studied^123–126^. This latter study would a priori shedlight on the role of the alkaline dopants on the local electronic & phonons configuration. Likewise, it might open new potential technological applications^127–130^ in the field of luminescence.

As a pre-conclusion, and in view of both the experimental observations and the additional computational investigations, it can be safely concluded that the observed significant reduction of the transition temperature is likely to be driven by the alkaline ions doping & not the interfacial strain/stress effect.

## Conclusion

This contribution reported on the thermochromic properties of a novel configuration consisting of V_2_O_5_/V/V_2_O_5_ stacks deposited onto borosilicate glass substrates by electron beam deposition. While the thickness of the V_2_O_5_ top and bottom layers were fixed, that of the intermediate V layer was varied within the range of 3–12 nm i.e. within the coalescence threshold of Vanadium. Such a system exhibited a crystal-clear thermochromic behaviour similar at a certain extent to that of pure VO_2_ but with far lower T_MiT_ and controllable/tunable thermochromic optical modulation in the NIR-IR; (∆T_TRANS_ = T_(T〈TMIT)_ − T_(T〉TMIT_). This latter tunability/control was validated via the intermediate V layer’s thickness. In addition to an elevated interfacial diffusion, a significant stress/strain in the V_2_O_5_/V/V_2_O_5_ stacks was observed. In a summary, the following findings can be highlighted.

1. The investigated V_2_O_5_/V/V_2_O_5_ stacks onto borosilicate glass substrate exhibited a net thermo-chromic response equivalent to that of pure VO_2_ thin films,
2. the investigated V_2_O_5_/V/V_2_O_5_ stacks onto borosilicate glass substrate exhibited a significantly low phase transition temperature of T_MiT_ = 27.5 & 37.5 ºC,
3. Such a significant lowering of T_MiT_ is caused by Alkaline ions doping originating from the borosilicate glass substrate
4. the investigated V_2_O_5_/V/V_2_O_5_ stacks experience a significant interfacial diffusion especially from Oxygen rich regions to Oxygen poor regions,
5. from stoichiometric viewpoint, the investigated V_2_O_5_/V/V_2_O_5_ stacks seem to be equivalent to an hypothetical V_x_O_y_ phase in between V_2_O_3_ & VO_2_,
6. the investigated V_2_O_5_/V/V_2_O_5_ stacks onto borosilicate glass substrate exhibited a net controllability of the NIR-IR optical modulation ∆T_TRANS_ versus the intermediate V layer’s thickness.

## Data Availability

The datasets used and/or analysed during the current study available from the corresponding authors on request.
